# Flexion First Balancer: description of new technique in TKA to reproduce joint line and pre-disease mechanical alignment

**DOI:** 10.1186/s40634-020-00241-x

**Published:** 2020-04-20

**Authors:** W. A. M. van Lieshout, K. L. M. Koenraadt, L. H. G. J. Elmans, R. C. I. van Geenen

**Affiliations:** 1 Dept. Orthopedic surgery, Amphia Hospital, Molengracht 21, 4818 CK Breda, The Netherlands; 2 Foundation for orthopedic research, care & education, Amphia Hospital, Breda, The Netherlands

**Keywords:** Knee osteoarthritis, Knee arthroplasty, Flexion first balancer, Alignment, Posterior condylar offset, Joint line

## Abstract

A considerable proportion of patients (19%) are dissatisfied after total knee arthroplasty (TKA). Possible factors contributing to this dissatisfaction are decreased posterior condylar offset (PCO) with subsequent joint line elevation, leading to mid-flexion instability. Secondly, the pre-disease mechanical alignment is changed into a neutral alignment. The Flexion First Balancer was developed to avoid these problems. This technique aims to maintain MCL isometry by restoring medial PCO and medial joint line to its pre-disease level. Also, to reconstruct the pre-disease mechanical alignment by adjusting the distal femoral angle. In this study we provide a detailed technical overview of the Flexion First Balancer technique.

## Background

Total knee arthroplasty (TKA) is an effective surgical intervention for patients with knee osteoarthritis [20]. In order to achieve optimal function and a stable TKA, a well-balanced knee in flexion and extension is essential. This is achieved by creating a symmetrical flexion and extension gap [10], restoring the native joint line [15] and reaching a neutral mechanical axis [6, 17]. The two main surgical philosophies in achieving a balanced neutrally aligned TKA are the measured resection technique and the balanced gap technique [4]. Both of these provide comparatively positive results and are widely accepted [19]. Despite this, 19% of the TKA patients are dissatisfied [2]. Multiple factors for dissatisfaction after TKA have been identified, both surgically [21] and psychologically [18].

A mechanical explanation for dissatisfaction after TKA might be that the current procedures do not reproduce the native joint kinematics and biomechanics. This is due to alterations made in the joint line height and the constitutional alignment. On average, the joint line is raised 3.0 mm after primary TKA [12]. The literature shows that more than 5 mm joint line elevation results in a worse clinical outcome [13]. This could perhaps be explained by the fact that elevation in joint line results in mid flexion instability [15]. This theory was reinforced by the findings of the study of Cross et al. which showed that by elevating the joint line the centre of rotation is placed more proximally and ventrally to its natural position, resulting in an unstable knee in the mid flexion range [3].

In a further attempt to improve functional outcomes, the concept of restoring constitutional alignment was introduced by Bellemans and colleagues. They showed that in the general population, 32% of men and 17% of women have a constitutional varus alignment [1]. Inevitably, correction of this pre-disease mechanical alignment during TKA to a neutral alignment requires ligament release(s). However, for these patients the most appropriate approach might be to restore their pre-disease mechanical alignment. With the current conventional techniques, the measured resection and the balanced gap techniques, constitutional alignment cannot be reproduced accurately, except by using navigation, robotic and/or patient specific instruments.

In an attempt to minimize mid-flexion instability and improve functional outcome, a new technique for TKA was developed: the Flexion First Balancer. This technique also offers the surgeon the ability to adjust the distal femoral angle based on the collateral ligament tension. Subsequently, the pre-disease mechanical alignment (i.e. soft tissue alignment) is restored, possibly resulting in a more ‘normal’ feeling knee. In this study a technical note is provided for this new technique.

### Aims of the flexion first balancer technique

**Fig. 1:**
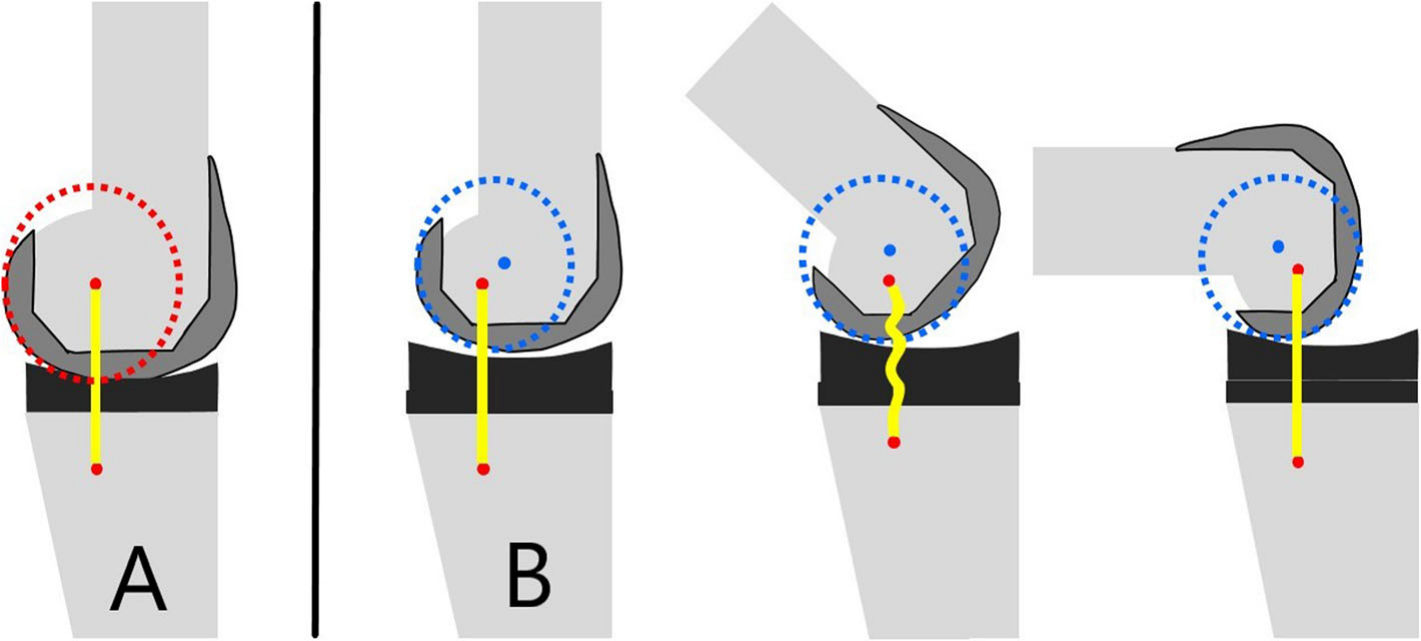
Mid-flexion instability after joint line elevation. Situation **a** represents a non-elevated joint line TKA, the center of rotation (red/blue dot) is restored by complete restoration of the posterior condylar offset and joint line height. The medial collateral ligament (marked yellow) will keep its isometry throughout the entire range of motion. Situation **b** represents an elevated joint line TKA with ticker insert to compensate. The axis of flexion (blue dot) no longer coincides with the MCL insertion (red dot). Therefore, the knee is stable in extension and 90° of flexion but laxity occurs in the mid-flexion range. The medial collateral ligament loses its isometric function in mid-flexion.

**Fig. 2:**
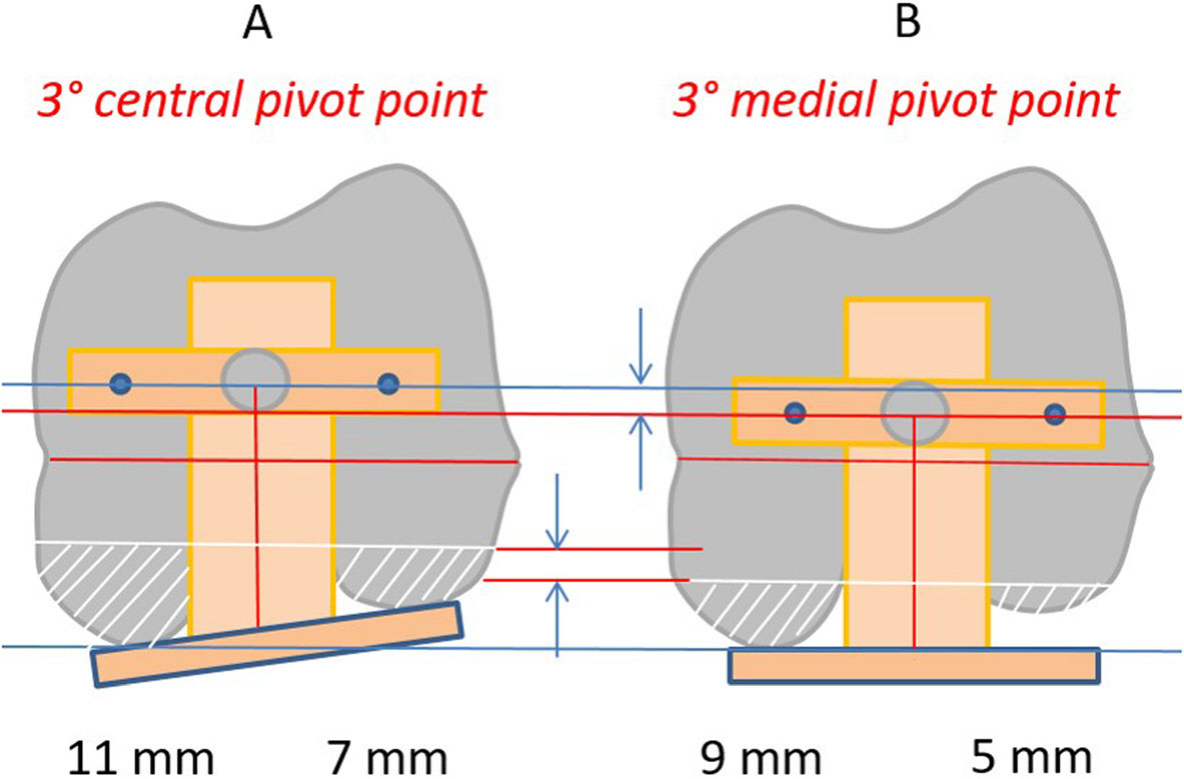
Differences in medial posterior condylar offset between conventional posterior reference technique and Flexion First Balancer technique. These figures show the differences in posterior condylar offset between conventional posterior referenced TKA systems (**a**) and the Flexion First Balancer (**b**). Both systems are set in 3° of exo-rotation for the posterior condyles (parallel to the trans-epicondylar axis). However, standard systems use a central pivot point and therefore averages the medial and lateral posterior condylar offset (PCO). As a result the medial PCO is not fully restored and this potentially leads to mid-flexion instability. The Flexion First Balancer technique pivots medially and thereby fully restores the medial PCO. The numbers in the bottom indicate the amount of resected bone from the medial and lateral PCO

The Flexion First Balancer technique has been developed with the aim of improving patient outcome. The philosophy behind this technique is that with preservation of the isometric function of the medial collateral ligament (MCL) the postoperative mid-flexion instability is minimized. It has been shown that a reduced posterior condylar offset (PCO) and subsequent joint line elevation will result in coronal laxity in the mid-flexion range since the axis for rotation of the knee is proximalized and ventralized (Fig. 1) [14]. The MCL isometry can only be preserved by fully restoring the medial PCO and the medial joint line height. By preservation of the medial anatomy of the knee (i.e. medial PCO and joint line) a balanced knee can be achieved and maintained throughout full range of motion. To achieve these goals, this technique uses the medial PCO as a reference for prosthesis positioning. By fully restoring the medial PCO, the natural medial joint line height can be recreated. This differs from conventional techniques where the average of the medial and lateral PCO is used, resulting in decrease of the medial PCO with subsequent mid-flexion instability (Fig. 2). When the joint line is set in flexion, the extension gap is matched to the flexion gap. By applying tension to the collateral ligaments with a balancer the distal femoral resection level and angle are determined. The Flexion First Balancer allows for restoration of the pre-disease mechanical varus or valgus alignment by adjusting the distal femoral angle. The distal femoral angle can be adjusted to balance the extension gap using wedges. This way natural soft tissue alignment is reconstructed and thereby also the pre-disease mechanical alignment. The restored alignment will presumably contribute to a more natural feeling of the TKA [22].

### Operative technique

A standard midline skin incision and medial para-patellar arthrotomy is performed. The first step is the tibial cut. An extra- or intramedullary resection guide is placed. The height of the cut should be set to six millimetres below the intact medial posterior cartilage of the tibia plateau. The cutting block is fixed and a perpendicular tibial cut is made. The technique aims to recreate the natural medial tibial slope of the patient. Generally, this can be achieved with a neutral cut to the mechanical axis of the tibia combined with the 4 degrees of slope of the CR insert. The thickness of the cut is slightly greater than in standard procedures in order to accommodate the implant without causing joint line elevation.

A straight intramedullary rod is now inserted into the femur and a standard distal femur cutting block is mounted to perform a preliminary five millimetre thick distal femur cut in 5^o^ valgus. The aim is to achieve a flat surface on the distal femoral condyles for the next step. The orthopaedic surgeon must avoid cutting in the trochlea. When the distal medial condyle is worn and no cartilage is present, we suggest only removing a four millimetre thick slice of bone from the medial condyle. In this manner, there is often only minimal contact on the lateral condyle.

**Fig. 3:**
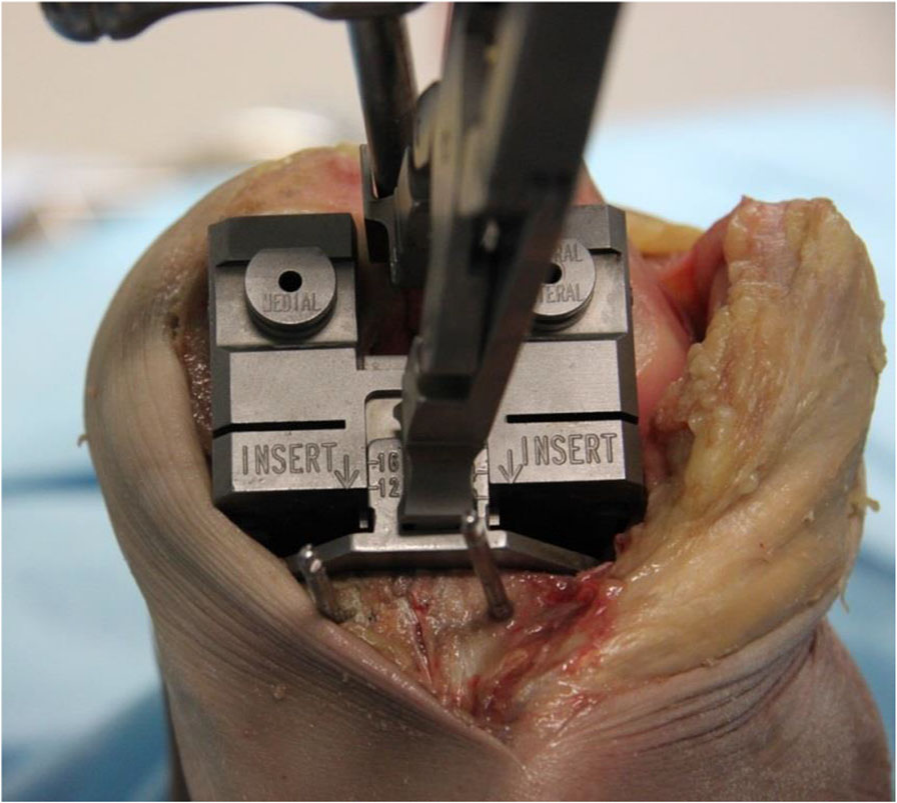
Flexion Balancer. The Flexion Balancer placed in a 90 degrees flexion setting with the distractor in situ to tension the flexion gap. The flexion gap should read at least 10 mm; in presented patient this gap reads 12 mm

The following step is the posterior condyle cut. Medial and lateral osteophytes are removed in this stage as these might interfere with ligament tension. In the native knee, in flexion, the lateral compartment is more lax than the medial compartment. This induces a medial pivot and allows for a more pronounced lateral roll back. When both collateral ligaments are tensioned using the FFB technique both ligaments are equally tensioned, the component is slightly externally rotated due to the lateral laxity, and these functional intercompartmental differences are lost. To preserve the correct rotation, the medial pivot and the slightly lax lateral compartment, the build-up of the PCO is reduced with 1 mm. Thus, a + 1 lateral bushing and the neutral medial bushing are used to ensure slightly more laxity in flexion of the lateral side. The flexion balancer is placed and tensioned with the distractor (Fig. 3). The flexion space is presented on the cutting block and should read at least ten millimetres to accommodate for 10 mm bearing thickness. Otherwise an additional tibial cut is required. With the distractor in place, drill pins are placed in the medial and lateral hole. The distractor is removed, and the femur is sized by referencing the anterior cortex on the lateral side. The pins are then removed. A slotted four-in-one cutting guide is placed and the posterior and anterior cuts can be made, the chamfer cuts are not performed yet. All instruments are subsequently removed. The flexion gap should now be rigorously cleaned out. Posterior osteophytes are removed with the use of a curved osteotome, designed to fit snugly behind the size matched osteophyte resection guide (if in between sizes, the smaller size is used). If a flexion contracture was present before surgery, a posterior capsular release should be performed at this stage. In case of a fixed valgus deformity we suggest a release of the postero-lateral capsule at this point. In doing so, extension and alignment can be achieved more easily. Subsequently, the flexion gap can be checked with the standard spacer blocks. The femoral spacer plate is then placed.

**Fig. 4:**
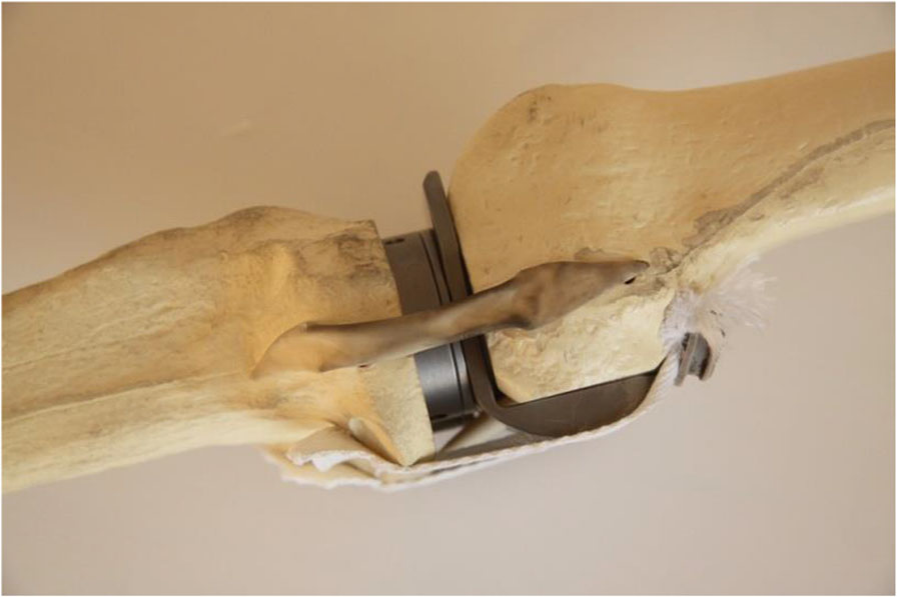
Tibial and femoral spacerplate. The femoral and tibial spacerplates are shown in situ to copy the flexion gap to the extension gap. The femoral spacerplate has a posterior condyle part which ensures a proper posterior capsule tightness

**Fig. 5:**
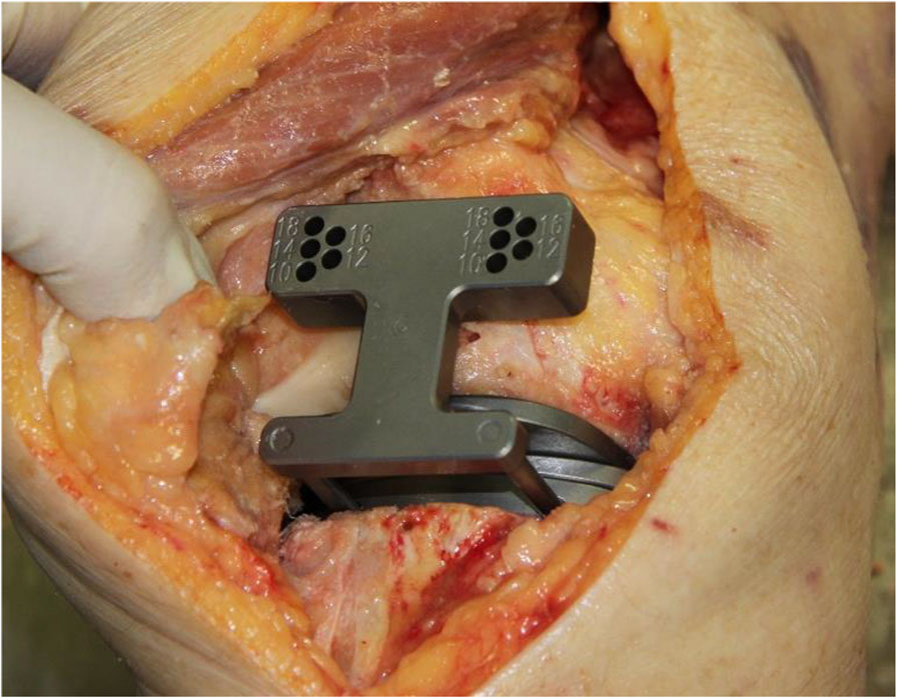
Drill guide for extension gap. The flexion gap is copied to the extension gap using the drill guide which is placed in the tibial spacerplate. Pins are subsequently drilled in the corresponding indicator holes in the femur

At this stage the flexion gap is set and the extension gap is matched to the flexion gap. The knee is extended with the tibia spacerplate and femoral spacerplate in situ. Spacer blocks are placed with increasing thickness until the extension gap is tight (Fig. 4). In case of a trapezoid extension gap the medial collateral ligament (MCL) and lateral collateral ligament (LCL) can be tensioned equally by using wedges. These come in 1, 2, 3 and 4 degrees of varus or valgus. By using wedged spacer blocks the valgus angle of the femur is adjusted to create a rectangular extension gap. When the extension gap is adequately balanced the drill guide is placed and the flexion gap is copied by drilling pins into the same indicators as previously measured (i.e. ten millimetres) (Fig. 5). After resection check the extension gap with the standard spacer blocks. All instruments are removed with exception of the drill pins. The distal femur cutting block is placed using the most proximal pinholes and the final distal femoral cut is made. With this step the femoral angle is adjusted to match the extension gap to the flexion gap without having to perform a partial release of the collateral ligaments. Then the chamfers cuts are finally made through the previously used slotted 4-in-1 cutting guide.

The orthopaedic surgeon now continues with the standard procedure to finish preparing the femur, tibia and patella. A cruciate retained (CR) type, Vanguard Complete Knee System (Biomet Orthopedics, Warsaw, IN, USA) implant is used. After placement of the components, the tension of the iliotibial band must be checked. Due to the build-up of the lateral condyle, the iliotibial band may be tight in slight flexion. If so, a (partial) release of the iliotibial band is advised.

## Discussion

This paper describes the new Flexion First Balancer technique, developed to restore the MCL isometry in order to achieve a stable knee throughout full range of motion. Key to achieving this goal is reconstruction of the medial anatomy of the knee. The Flexion First Balancer technique therefore uses the medial PCO as a reference. Hereby, the medial joint line is preserved, and the flexion gap is set. Now the extension gap is matched to the flexion gap. By reconstructing the medial PCO and joint line to their pre-disease level the isometric function of the MCL is preserved.

The philosophy of matching the extension gap to the flexion gap has been described in the past by pioneers in TKA surgery like Freeman and Insall. However, in the 70s, there was not the wide availability of the many different sizes of prosthetics we have today. Frequently the PCO was decreased to fit the femoral component without notching or overstuffing the patella-femoral joint. As a result the joint line was elevated, with its inherent risks [7, 15]. The flexion first technique was therefore replaced with the extension gap first technique to gain control over the joint line position. Today, the quality and availability of prosthetics and instruments has improved enormously. As described above, the Flexion First Balancer provides an easy and reproducible technique to reconstruct the medial anatomy of the knee and thereby maintain the MCL isometry.

Beside preservation of MCL isometry, the Flexion First Balancer also balances the knee through an adjustable distal femoral angle using the collateral ligaments tension in extension, subsequently reconstructing pre-disease mechanical alignment. Previously, other techniques have been reported to achieve the same goal. Howell and colleagues described techniques to restore constitutional alignment by adjusting the tibial cut leading to a restoration in kinematical alignment [9]. However, this technique has some disadvantages. The tibial resection needs to be adjusted to match the varus in the proximal tibia. This leads to an increased valgus angle for the femoral component, which should be parallel to the femoral flexion-extension axis. These alterations might affect patellar tracking, as the femoral component is not designed for this alternative position. Secondly, moving away from a perpendicular tibia cut is correlated with high failure rates due to tibial loosening and/or ligament instability [16]. There is no consensus concerning which method is ideal for the patient. However, the Flexion First Balancer provides a reproducible and easy technique to recreate the natural soft tissue alignment by adjusting the distal femoral angle with a neutral tibia plateau without compromising patellar tracking. More importantly, since this new technique uses a perpendicular tibial cut, the risks of increased tibial loosening can, via this method, be avoided [16].

New techniques carry the risk of introducing new negative side effects. Theoretically, a potential negative side effect could be lateral tightness of the knee. Due to the build-up of the lateral posterior condyle this could result in a tight flexion gap on the lateral side. This phenomenon also occurs in normal TKA techniques, however in the FFB technique this build-up is increased and the joint line on the lateral side is more distalized compared to normal TKA techniques. This could result in a tighter lateral compartment, especially in mid-flexion when the iliotibial band (ITB) crosses the lateral condyle at 30 degrees of flexion [5]. Theoretically resulting in pain on the lateral side of the knee and difficulties in bending the knee as is seen in the iliotibial band syndrome. Therefore, we suggest checking the lateral tightness per-operatively and if applicable a (partial) release of the ITB should be performed. A potential complication is that by changing the valgus angle of the femur, a HKA angle is created that is outside the presumed safe zone of − 3 to + 3 degrees to the neutral mechanical axis. Traditionally this was considered an important aspect of TKA. However, present literature is less convincing. Hadi et al. performed a review of the literature and showed no correlation between outliers in HKA in the coronal plane after TKA and revision rates [8].

A limitation of the current study is that it does not provide patient data to support the presumed benefits of the Flexion First Balancer method. However, another recent publication discussed PROMS and patient complication rates in a comparison of the standard MR procedure and this new technique [11]. Therefore, the main focus of this article is to provide a clear description of the technical application of the Flexion First Balancer method.

## Conclusions

In conclusion the Flexion First Balancer technique offers the surgeon the ability to recreate the medial anatomy of the native knee in order to maintain the isometry of the medial collateral ligament, which is considered essential to prevent mid-flexion laxity.

## Data Availability

Not applicable

## Abbreviations

CR: Cruciate retained
FFB: Flexion First Balancer
ITB: Iliotibial Band
LCL: Lateral Collateral Ligament
MCL: Medial Collateral Ligament
OA: Osteoarthritis
PCO: Posterior Condylar Offset
TKA: Total Knee Arthroplasty
